# The noseleaf of *Rhinolophus formosae* focuses the Frequency Modulated (FM) component of the calls

**DOI:** 10.3389/fphys.2013.00191

**Published:** 2013-07-19

**Authors:** Dieter Vanderelst, Ya-Fu Lee, Inga Geipel, Elisabeth K. V. Kalko, Yen-Min Kuo, Herbert Peremans

**Affiliations:** ^1^Active Perception Lab, University AntwerpAntwerp, Belgium; ^2^Department of Life Sciences, National Cheng Kung UniversityTainan, Taiwan; ^3^Institute of Biodiversity, National Cheng Kung UniversityTainan, Taiwan; ^4^Institute of Experimental Ecology, University of UlmUlm, Germany; ^5^Smithsonian Tropical Research Institute, Barro Colorado IslandBalboa, Panama

**Keywords:** rhinolophus, chiroptera, emission, formosae, ranging, noseleaf, furrows, lappets

## Abstract

Bats of the family Rhinolophidae emit their echolocation calls through their nostrils and feature elaborate noseleaves shaping the directionality of the emissions. The calls of these bats consist of a long constant-frequency component preceded and/or followed by short frequency-modulated sweeps. While Rhinolophidae are known for their physiological specializations for processing the constant frequency part of the calls, previous evidence suggests that the noseleaves of these animals are tuned to the frequencies in the frequency modulated components of the calls. In this paper, we seek further support for this hypothesis by simulating the emission beam pattern of the bat *Rhinolophus formosae*. Filling the furrows of lancet and removing the basal lappets (i.e., two flaps on the noseleaf) we find that these conspicuous features of the noseleaf focus the emitted energy mostly for frequencies in the frequency-modulated components. Based on the assumption that this component of the call is used by the bats for ranging, we develop a qualitative model to assess the increase in performance due to the furrows and/or the lappets. The model confirms that both structures decrease the ambiguity in selecting relevant targets for ranging. The lappets and the furrows shape the emission beam for different spatial regions and frequency ranges. Therefore, we conclude that the presented evidence is in line with the hypothesis that different parts of the noseleaves of Rhinolophidae are tuned to different frequency ranges with at least some of the most conspicuous ones being tuned to the frequency modulated components of the calls—thus yielding strong evidence for the sensory importance of the component.

## Introduction

Various bat species emit their echolocation calls through their nostrils (Nowak, 1994). In these species, the nostrils are often surrounded by leaf- or spear-like structures called noseleaves. Noseleaves have been shown in experiments (Hartley and Suthers, 1987) and in acoustic simulations (Vanderelst et al., 2010) to act as baffle and to focus the emission beams of bats. The most elaborate noseleaves are found in the Horseshoe bats (Rhinolophidae) and the Old World Leaf-Nosed bats (Hipposideridae). Bats of both families emit echolocation pulses consisting mostly of a single narrow constant frequency (CF) component that is often preceded and/or followed by a very short frequency modulated (FM) component (See Jones and Teeling, 2006; Schnitzler and Denzinger, 2011 for a review).

**Figure 1:**
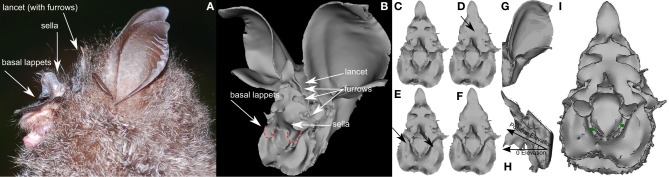
**Rendering of the 3D models of the noseleaf and pinna of *Rhinolophus formosae* used to simulate the emission and hearing beam patterns. (A)** Photo of *R. formosae* (courtesy of Chun-Wei Hsie). **(B)** Rendering of complete head model from which the noseleaf model **(C–F)** and the pinna model **(G)** were derived. In panels **(A,B)** the different parts of the noseleaf have been indicated. In panel **(B)** the basal lappets have been circled. **(C)** Original noseleaf model. **(D)** Model with filled furrows (see arrow). **(E)** Noseleaf model with removed basal lappets (see arrows). **(F)** Noseleaf model with both filled furrows and removed basal lappets. **(G)** Pinna model. Note that the rendered pictures **(C–F)** and **(H)** are not aligned but have been rotated to facilitate viewing of the features of the noseleaf. **(H)** Model indicating the reference position of the noseleaf model. **(I)** Rendering of the noseleaf with the position of the virtual receivers indicated by green dots. The supporting material provides movies of the rotating models.

Zhuang and Muller (2006) argued based on acoustic simulations, that the furrows (see Figure 1) on the noseleaf of the Rufous Horseshoe Bat *Rhinolophus rouxii* function as resonance cavities de-focusing the emission beam at the lowest frequencies contained in the FM component of the call. Recently, we attempted to replicate this study but found only partial agreement. In accordance with Zhuang and Muller (2006), the results of our study indicate that the furrows affects the emission beam most for frequencies in the FM component of the echolocation call. However, we found that the noseleaf furrows, in accordance with the functionality of noseleaves in other bat species (Hartley and Suthers, 1987; Vanderelst, De Mey, Peremans, Geipel, Kalko, and Firzlaff, 2010), aid in focusing the emission beam (Vanderelst, Jonas, and Herbert, 2012). Apart from the disagreement of the two studies about the specific effect of the furrows (i.e., focussing vs. de-focussing), both studies found that the noseleaf furrows of *R. rouxii* act on the emission beam at the frequency range of the FM part of the call. These results suggest that the lancets of Rhinolophids are morphological structures adapted to the FM component of the calls yielding direct evidence for the importance of the FM component for the sonar system of these bats. This is somewhat unexpected, as Rhinolophids are otherwise known for featuring a wide range of anatomical and physiological specializations tuned to processing the CF component of their call (Reviewed in Schnitzler and Denzinger, 2011).

As the evaluation of the function of noseleaf furrows was performed only for a single species, the generality of this result remains to be confirmed. In this paper, we investigate the effect of the furrows in the noseleaf of the Formosan Wooly Horseshoe Bat *Rhinolophus formosae* (Sanborn, 1939). Specifically, we seek further support for the hypothesis that the furrows in Rhinolophids play a dominant role for the FM frequency range of an emitted call. In addition to furrows, the noseleaf of *R. formosae* also features two flaps (the base of the sella has a pair of circular basal lappets, see Figure 1) partially covering the nostrils. We hypothesize that these flaps aid in focusing the emission beam by interacting with the emitted sound field. Furthermore, we suggest that if these flaps influence the sound field primarily at the FM frequencies, it provides further support for the hypothesis that particular noseleaf structures of Rhinolophids are not tuned to the CF but to the FM component. Finally, this paper introduces a formal model to quantify the functional relevance of noseleaf structures in bats, e.g., furrows and basal lappets in the case of *R. formosae*, based on the current understanding of the function of the FM component.

## Methods

### Model construction and simulation

We used the Boundary Element Method (BEM) to simulate the directionality of the echolocation system of *R. formosae*. As we have reported in detail on this method and its validation elsewhere (De Mey et al., 2008; Vanderelst et al., 2012), we will only describe the simulation method briefly. Using BEM to simulate the sound field around a bat's head requires the construction of a detailed mesh model of the head morphology. A single specimen of *R. formosae* was collected by mist-netting in Kenting, Taiwan, in 2010. We first preserved the specimen in 95% ethanol, and later in a sealed and air-proofed specimen box, well cushioned with wet cotton cloth during shipping to maintain constant humidity levels and preserve the natural shape of the outer ears and the facial structures. The head of the specimen of *R. formosae* was scanned with a MicroCT machine using a resolution of 70 μm. After reconstructing the shadow images, an initial mesh model of the complete head was obtained using a set of standard biomedical imaging tools (see Figure 1 for renderings of the models).

Current computational facilities allow to simulate models containing up to 35,000 triangles. The noseleaf of *R. formosae* is a very complex structure consisting of two rows of furrows and basal lappets overhanging both nostrils. Furthermore, in comparison to most other echolocating bats, *R. formosae* is a relatively large species, with adults averaging around 21 g in body mass and 58 mm in forearm length (Lee et al., 2012). Hence, to construct a model of sufficient detail of the noseleaf, we made a separate model of the noseleaf to simulate the emission directionality of *R. formosae*. As the pinna is also quite large compared to that of most other echolocating bats, we again made a separate model of the left pinna of the bat. The mirrored left ear (and its sensitivity pattern) was used as a replacement for the right ear.

The initial noseleaf and pinna models were subjected to several rounds of smoothing and remeshing to reduce the number of triangles in the models to about 30,000. The maximum edge length of the final models was 0.6 mm. At 80 kHz, the highest frequency employed in the simulations, an edge length of 0.6 mm results in a sampling of 7 nodes per wavelength (4.2 mm) which was sufficient to obtain stable simulation results. From the original noseleaf model, two additional models were derived. In one model we filled the furrows in the upper part of the noseleaf , whereas in the other noseleaf model, we removed the basal lappets of the sella that overhang the nostrils (see Figure 1 for renderings and the supporting material for movies of the models).

To simulate the emission beam pattern we placed a virtual receiver in both nostrils of the noseleaf model (see Figure 1I). Placing receivers in the noseleaf model to simulate the emission beam pattern is warranted by the reciprocity principle (Pierce et al., 2008) and enhances numerical stability of the simulations (Moller and Cutanda Henriquez, 2009). To obtain the emission beam pattern, the complex sound fields of the left and the right nostril are summed and the magnitude of this sum is reported. Simulating the directional hearing sensitivity was done by placing four virtual receivers in the ear canal of the pinna model. Virtual omnidirectional sources are placed on a sphere with a diameter of 1 m around the bat noseleaf model. The sources are spaced 2.5° apart covering −90 to 90° in both azimuth and elevation (i.e., 5329 sources). Placing the sources in this regular configuration allows for easy preprocessing of the data. This configuration, however, does not sample the sound field on the sphere uniformly. Therefore, we resampled both the emitted sound field and the hearing directionality at 528 equally spaced positions during the processing of the data using the Recursive Zonal Equal Area Sphere Partitioning Toolbox (Leopardi, 2006). In processing the emission beam pattern, we assume that all the emitted sound energy stays within the frontal hemisphere, i.e., negligible amounts of energy are radiated backward, requiring the normalization of the emission beam patterns of the bats per frequency *f*,

(1) $$\int_{\Omega }p_{f,\;\phi ,\;\theta }^{2}\cdot d\phi d\theta =1$$

with *p* denoting the magnitude of the emission strength for frequency *f* in direction (azimuth = ϕ, elevation = θ) and Ω the frontal hemisphere.

To assess the roles of the furrows and the flaps in focusing the emission beam, we calculate the average gain ḡ for the normalized emission beam patterns,

(2) $$\bar{g}=\frac{\int_{\Omega }g_{f,\;\phi ,\;\theta }}{\Omega }$$

with the gain for a particular direction and frequency given by

(3) $$g_{f,\;\phi ,\;\theta }=10\cdot \log_{10}\frac{p_{f,\;\phi ,\;\theta }^{2}}{\max_{\Omega }p_{f,\phi ,\theta }^{2}}$$

In accordance with Schnitzler and Grinnell (1977) and Firzlaff and Schuller (2004), the model was oriented such that the horseshoe of the noseleaf was vertical (see Figure 1H for an illustration of the coordinate system used in this paper).

### Call recordings

In order to asses the frequency range of the FM components of the calls of *R. formosae*, recordings were collected from 7 individual bats and 159 calls were extracted and analyzed. Echolocation calls of *R. formosae* individuals were recorded every evening between the 5th and 12th of October 2010, in the Guijijaou Experimental Forest and Hengchun Tropical Botanical Garden (HTBG, 120°48′*E*, 20°58′*N*, ca. 450 ha in area and 200–300 m in elevation; Taiwan Forestry Research Institute), Kenting, Taiwan. Recordings were started around sunset in the prime activity period of the bats (Lee et al., 2012), and lasted until around 23:00. Echolocation calls were recorded using a condenser microphone (microphone capsule CM16, CMPA preamplifier unit, Avisoft Bioacoustics, Berlin, Germany) and digitized with a real time ultrasound acquisition board (UltraSoundGate 116, Avisoft Bioacoustics, Germany; 375 kHz sampling rate, 16 bit resolution) connected via USB port to a laptop computer (Eee PC, ASUS, Taiwan). We walked along different trails in the forest and around different edge or open sites to avoid sampling the same individuals.

## Results

### Acoustic recordings

**Figure 2:**
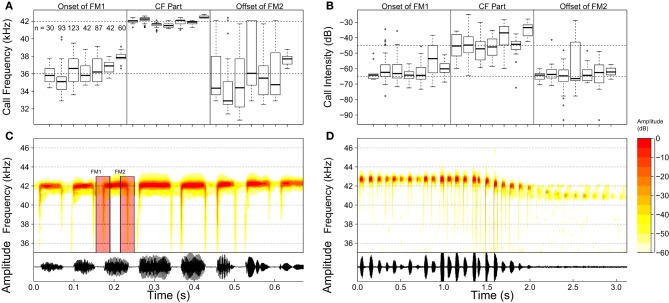
**(A)** Boxplots for the frequency of the FM and CF parts for each of the seven bat specimens whose calls were analyzed. Open circles denote outliers defined as data points that lie below *Q*_1_ − 1.5 × (*Q*_3_ − *Q*_1_) or above *Q*_3_ + 1.5 ×(*Q*_3_ − *Q*_1_) (with *Q*_*n*_ the *n*th quartile). The number of calls analyzed for each individual are displayed in the leftmost section of the panel. **(B)** Similar but for call intensity. **(C,D)** Two spectrograms of call sequences of *Rhinolophus formosae*. **(C)** calls of a perched bat. The frequency modulated parts (FM_1_ and FM_2_) of one of the calls are indicated. **(D)** calls of a perched bat taking off. Only the strongest first overtone is shown in this figure.

In total, 159 calls of high quality from seven individuals were chosen to be analyzed (Spectrograms were calculated using a 256 sample FFT with 75% overlap and Hanning windowing). Figure 2 displays the spectrograms of two call sequences of *R. formosa.* Only 4% of the analyzed calls had neither a leading (FM_1_) nor a trailing (FM_2_) FM part. Of all calls (*n* = 159), 18% missed a leading (FM_1_) and 10% a trailing (FM_2_) FM component. Where present, the lowest frequency of the FM parts was extracted using AviSoft SaSLab Pro (Raimund Specht, Berlin). In addition, the frequencies of the CF part and the intensity of both FM and CF parts were registered. The resulting data for the seven bats are plotted in Figure 2. The FM parts were 15–20 dB weaker than the CF parts. Our acoustic recordings indicate that *R. formosae* uses calls with a CF of about 43 kHz and FM parts spanning from 36 to 43 kHz. This frequency range covers the first overtone of the bat's calls. The other harmonics were detectible in the recordings but were typically 30–40 dB weaker.

### Simulations

**Figure 3:**
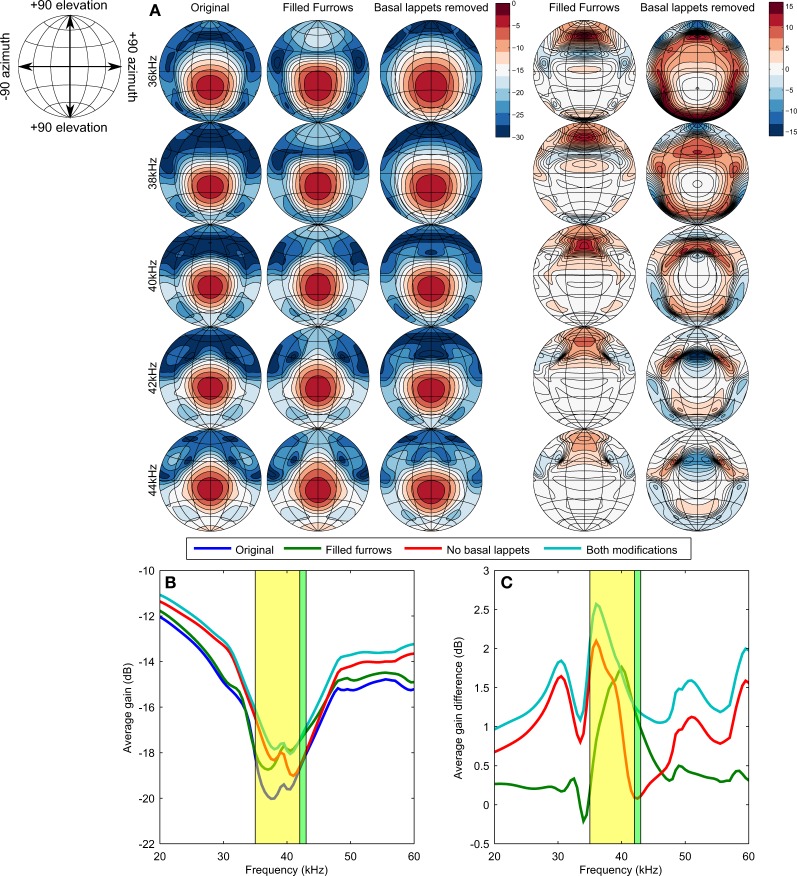
**(A)** Simulated emission beam patterns for the original model and the models with filled furrows or without flaps (contours are spaced 3 dB apart). The two right most columns show the difference in gain (ḡ) between the original model and the two altered models (contours are spaced 1 dB apart). **(B)** Average gain of the emission beam pattern as function of frequency. The frequency ranges of both the FM and the CF components are shaded in yellow and green, respectively. The maps in panels **(A,B)** are Lambert azimuthal equal-area projections centered around zero azimuth and elevation. The parallels and meridians are 30° apart. See the top left inset for the definition of the axes. **(C)** similar as **(B)** but showing the average gain difference with the original model as a function of frequency.

Figure 3 displays the simulated emission beam patterns for selected frequencies and the different noseleaf models. The emission beam consists of a single mainlobe located little below the horizontal plane (i.e., around −15° elevation). Filling the furrows increases the gain of the emission beam pattern for high elevation positions (around 60° elevation). The flaps reduce the gain of the emission beam pattern in a circular area around the mainlobe. The average gain of the emission pattern shows a global minimum in the frequency range coinciding with that of the calls of *R. formosae*. Removing either the basal lappets or filling the furrows results in an increase in the average gain (see Equation 2) and thus a loss in directivity. The effect of filling the furrows and removing the lappets on the average gain is largest in the frequency range of the calls of the bat. However, the largest effect is not found around the CF frequency (42–43 kHz) but in the frequency range of the FM part of the call. The effect of filling the furrows is largest for 40 kHz. For the lappets, the effect is largest for 36 kHz.

The simulation results were confirmed by acoustic measurements using 3D printed versions of the original noseleaf model and the model without lappets and filled furrows. In the measurements, the structures also focussed the beam most strongly around 36 kHz (results provided as supporting material).

### Quantification of the noseleaf functionality

The finding that characteristic substructures of the noseleaf of *R. formosae* have the largest acoustic effects at the frequencies of the FM part of the calls suggests that the FM component is an integral and important part of the calls of CF/FM bats. These bats are assumed to detect, identify and locate prey based on the frequency modulations of the CF component of the echo caused by fluttering prey. Listening for frequency modulations in the echo using a highly specialized hearing apparatus makes these bats highly robust with respect to clutter echoes (Schnitzler and Denzinger, 2011). Echoes originating from stationary vegetation can be effectively filtered out by the hearing apparatus and the Doppler shifted parts of the echo contain sufficient localization information (Vanderelst et al., 2011). Conversely, the bat has no mechanism to reliably filter out any FM echoes generated by clutter objects. Hence, featuring morphological adaptations to focus the beam in the FM part of calls makes sense considering the echolocation strategies of these CF/FM bats. As reviewed by Schnitzler and Denzinger (2011), the FM parts of the calls of Rhinolophus bats are assumed to be used predominantly for measuring range. Therefore, we developed a model quantifying the effectivity of clutter rejection of the lappets and furrows in a ranging task.

We will assume that ranging only requires the FM part of the echo from a target object. From radar theory (Skolnik, 1980) it is known that both detection probability and ranging accuracy depend upon the energy in the received signal. Hence, we assume the bat estimates the energy in this FM part of the echo *E*,

(4) $$E=10\cdot \log_{10}\int_{f}p_{f}^{2}$$

with *p*_*f*_ the magnitude of the Fourier transform of the sound pressure level at frequency *f*. Having reduced each echo to a scalar energy estimate, the simplest strategy for the bat is to interpret the echo with the highest energy as coming from the object onto which it has centered its beam, which coincides more or less with the flight direction of the bat. Such a mechanism will result in selecting the correct echo most of the time. However, the mechanism breaks down in the presence of strong clutter reflectors. The probability of selecting the incorrect clutter echo *C*_ϕ, θ,_ originating from azimuth ϕ and elevation θ as the one coming from the target object can be written as,

(5) $$P(E_{T,\;H}<E_{C,\;H}|C_{\phi ,\;\theta })=1-\int_{-\infty }^{0}L\cdot \int_{-\infty }^{0}R$$

The energy of both the target and the clutter echo depend on the spatial sensitivity *H* of the bat (i.e., combination of emission beam pattern and auditory spatial sensitivity). In Equation (5), *L* and *R* denote the normally distributed energy of the clutter echo arriving at the left and right ear, respectively. This is, *L* = *N(Ê_ϕ, θ_|l*, σ) and *R* = *N*(*Ê*_ϕ, θ_|*r*,σ) with *Ê*_*l*, ϕ, θ_ and *Ê*_*r*, ϕ, θ_ the expected energy of a clutter echo coming from azimuth ϕ and elevation θ and arriving at the left and the right ear, respectively, given the spatial sensitivity of the left (*l*) or the right (*r*) ear. In Equation (5), we assume that the reflector strength of objects in the environment is normally distributed with a standard deviation given by σ.

Using Equation (5), we can calculate, for each of the noseleaf models and their respective emission beam patterns, the probability that an echo arriving from a given location will interfere with the correct selection of the FM echo coming from the target for a given value of σ. In addition, we calculated the difference in the probabilities for the models in which the furrows were filled or the flaps were removed.

Selecting values for σ should preferably be done based on empirical measurements of the variation in the energy of echoes returning from a large sample of different plants. However, to the best of our knowledge, no such estimates have been published. Therefore, we extrapolate the value for σ from measurements we collected earlier as well as data provided by Ralph Simon (University Ulm). This data set consists of echoes collected from fluttering insects and a number of flowers from different aspect angles. Earlier we showed, based on these data, that the variation in energy for a narrow frequency band could be adequately modeled using a normal distribution with a standard deviation of about 10 dB (see supplementary material of the paper by Reijniers et al., 2010). The variation in energy of a broadband echo can be approximated based on this data by making an adequate assumption about the number of independent frequency channels an echo will stimulate in the cochlea of the bat. This can be estimated using the Equivalent Rectangular Bandwidth (ERB). Extrapolating the formula for calculating ERB values given in Moore and Glasberg (1983). for the bat's frequency range, a frequency channel with center frequency *f* = 40 kHz corresponds with an ERB value of 4.3 kHz. This implies that the frequency range of the FM component (about 7 kHz) can be modeled by about 2 independent frequency channels. Assuming 2 independent frequency channels and a standard deviation of 10 dB per frequency channel yields a standard deviation of about 14 dB for the energy of an echo. Based on this extrapolation, we evaluate the model for a wide range of σ values around 14 dB, i.c. from 10 to 25 dB.

**Figure 4:**
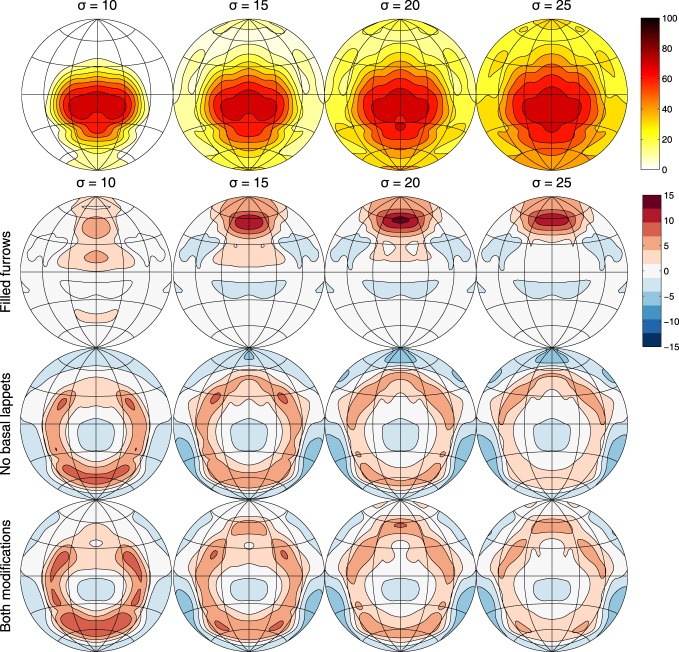
**Row 1:** The probability of the energy of an FM echo coming from a clutter object being higher than an echo coming from the target position as a function of azimuth and elevation (Contours spaced 10% apart) and for different values of σ. As specified in the main text, the reflector strength of objects in the environment is assumed to be normally distributed. The standard deviation of this distribution is given by σ. These plots show the area in which confusion between the target echo and an interfering echo is most likely. **Rows 2–4:** the gain in the probability of confusion by removing either furrows, basal lappets or both (Contours spaced 2% apart). Plots show the averages across the frequency range of the calls (i.e., from 36 to 43 kHz).

Figure 4 displays the interference probability *P*(*E_T, H_ < E_C, H_|C*_ϕ, θ_) as a function of azimuth and elevation as well as the increase in error probability associated with having either furrows, flaps or both structures removed. Irrespective of the value of σ, the probability of confusing a non-target FM echo with the FM echo is the largest in a ellipsoid area around azimuth 0 and elevation −15°. The furrows reduce the probability confusing the target and non-target echo mostly in an area between 0 and +30° azimuth. The flaps reduce the confusion in a circular area around −15° elevation.

As demonstrated above, the furrows and the basal lappets alter the emission beam pattern mostly in a frequency range coinciding with the FM part of the call. To confirm that the furrows and the basal lappets cause the largest reduction in the probability of confusion in the same frequency range, we calculate the expected angular error due to the removal of the basal lappets and the filling of the furrows across the frontal hemisphere for a range of frequencies and values of σ. In particular, we calculate the increase in angular error resulting from the removal of the furrows and the flaps for frequency ranges given by [*f*_low_, *f*_low_ + Δ*f*] with *f*_low_ ranging from 30 to 50 kHz and Δ*f* set to 7 kHz (the range spanned by the FM component). The increase in error is given by the expected distance in degrees *E*(ζ) between the strongest echo and the target echo using Equation (6). In this equation, *G*(ϕ, θ) gives the arc length in degrees between the direction of the target echo and the direction of the interfering echo.

(6) $$E(\zeta )=\int^{\phi ,\;\theta }P(E_{T,\;H}<E_{C,\;H}|C_{\phi ,\;\theta })\times G(\phi ,\;\theta )$$

**Figure 5:**
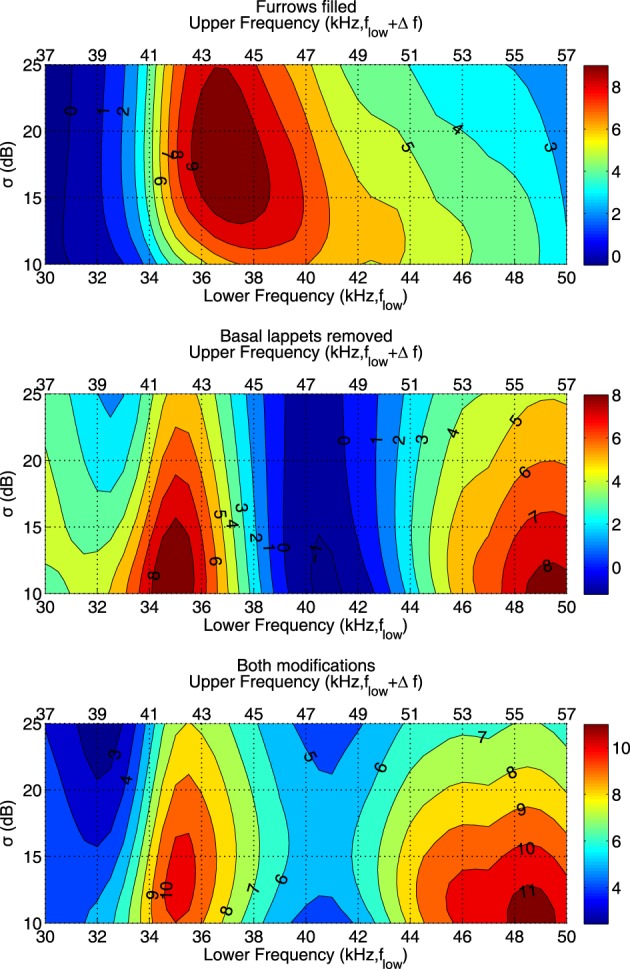
**The expected angular error **E**(ζ)(in degrees) between the strongest echo and the target echo averaged across the frontal hemisphere for different frequency ranges and values of σ and different noseleaf models.** The lower x-axis depicts the lower frequency of the interval. The upper x-axis shows the upper frequency.

Figure 5 shows that the effect of the both the furrows and the lappets is largest in a frequency range coinciding with the FM range. In agreement with the effects of both structures on the average gain, the effect of the furrows is maximal for a frequency range starting at a somewhat higher frequency than the effect of the basal lappets. The effect of the furrows is maximal for the range (37, 44 kHz) while the effect of the lappets is largest for the frequency range (35, 42 kHz).

## Conclusion

Simulating the effect of both the furrows and the flaps of the noseleaf of *R. formosae* on the emission pattern, we find that both structures aid in focusing the emission beam. The largest effect of these structures is not found around the CF frequency but in the frequency range of the FM parts of the calls. Interestingly, the frequency at which the effects of each structure is largest differs: the lappets have the largest impact at somewhat lower frequencies than the furrows. However, the structures are not only complementary in terms of frequency. The effects of both structures are also spatially complementary as the furrows influence the beam mostly for high elevations while the lappets focus the beam in a circular area around the main beam.

Currently, the best supported hypothesis about the functionality of the FM component is that it is used in ranging. The model presented in the paper suggests that the lappets and furrows increase the bat's ranging accuracy by suppressing echoes coming from peripheral targets. Moreover, the model showed that the angular error in selecting the target echo is reduced most efficiently for frequency ranges coinciding with the FM component.

In summary, the evidence presented here and elsewhere (Zhuang and Muller, 2006; Vanderelst et al., 2012) suggests a division of labor between different substructures of the noseleaves of Rhinolophidae with various morphological structures shaping the soundfield at different frequencies. Moreover, at least some of the most conspicuous features seem to be tuned to the FM component.

### Conflict of interest statement

The authors declare that the research was conducted in the absence of any commercial or financial relationships that could be construed as a potential conflict of interest.
