# Supplementary material for: The noseleaf of Rhinolophus formosae focuses the Frequency Modulated (FM) component of the calls
Source: Front Physiol. 2013 Jul 19;4:191. doi: 10.3389/fphys.2013.00191 (PMC3715718; doi:10.3389/fphys.2013.00191)
Supplement: Supplementary file 6 [file DataSheet1.PDF]

The noseleaf of Rhinolophus formosae focuses the Frequency Modulated (FM) component of the calls: Supplementary Material

We performed a series of measurements on 3D printed versions of the models to validate the simulation results. Using a rapid prototyping machine we manufactured printed versions of the original noseleaf model and of the noseleaf model in which the flaps were removed and the furrows filled (figure 1). The models were fitted with two holes in which Knowles FG Series microphones were inserted. The holes were positioned such that the centers of the microphones coincided with the positions of the virtual receivers in the simulations.

The models were fixated on a laboratory stand in front of a robot arm equipped with a Polaroid ultrasonic emitter (see figure 2). The robot arm was used to ensonify both models from 250 equally spaced directions between -60 and + 60 degrees azimuth and -75 and +75 degrees elevation. The signal used was a sweep from 80 to 30 kHz with a duration of 0.5 ms. The distance between the Polaroid and the model was 250 mm. The microphone signals were amplified and recorded at a sample rate of 250 kHz.

To obtain the emission pattern, the recorded signals in both nostrils were summed. For each of the 250 directions, the spectrum was calculated from the summed signal for a number of frequencies of interest using the Goertzel algorithm. For each model we collected two sets of data. The microphone in the left nostril was inserted into the right nostril and vice versa for the second measurement to balance any differences in frequency response between the microphones. Here we report on the average of both measurements. Moreover, we made the measured emission patterns symmetrical by taking the average of the measured pattern and the same mirrored pattern.

The results are depicted in figures 2 and 3. Although we only measured part of the frontal hemisphere, the measurements revealed the same focusing effect of the flaps and the furrows as found in the simulations. As shown in figure 4, the effect of the furrows and lappets was maximal around 36 kHz, i.e. the same frequency at which the effects in the simulations were maximal. The average effect of the furrows and lappets was somewhat smaller in the measurements than in the simulations because large effects were found in the periphery which was not included in the measurements.

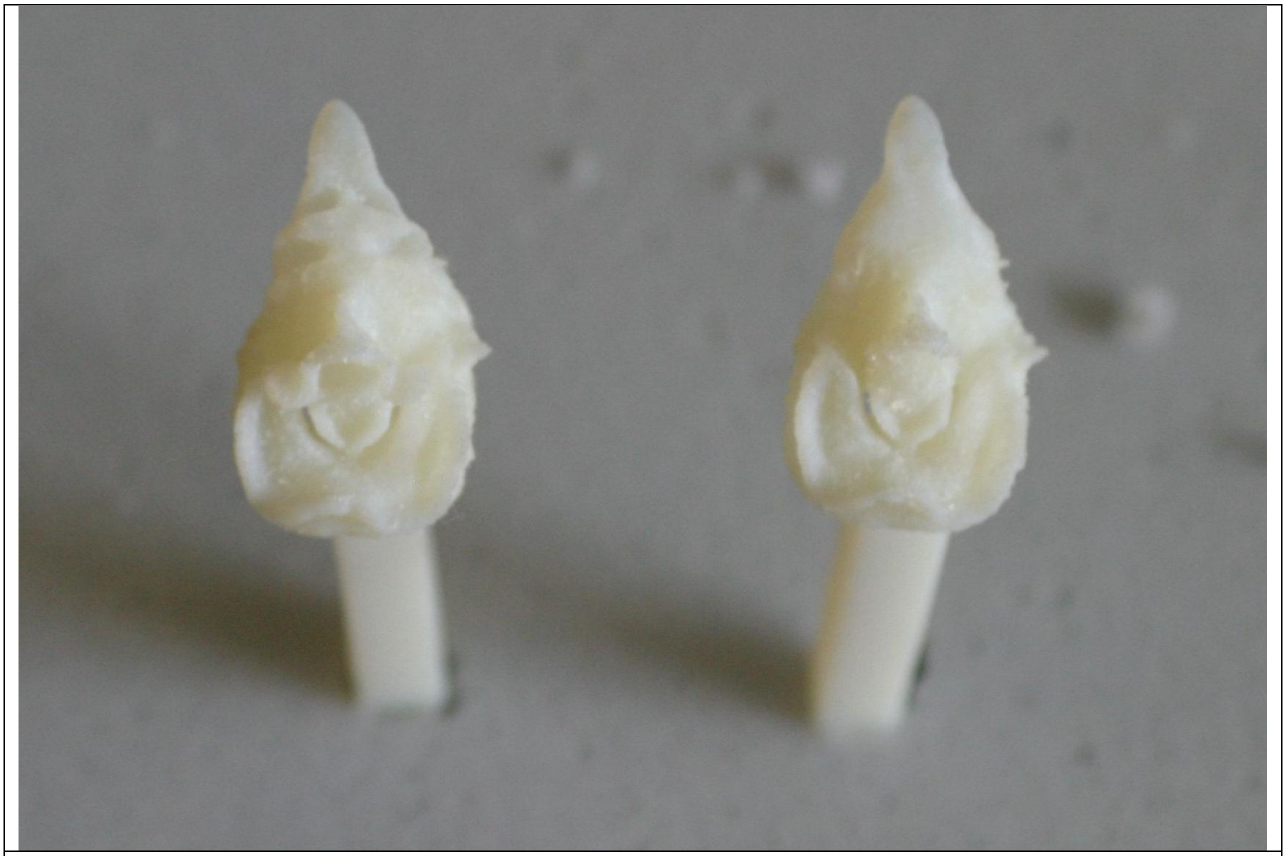

Figure 1: Picture of the 3D printed models. Left: the intact noseleaf, Right: the noseleaf model in which the furrows were filled and the flaps removed.

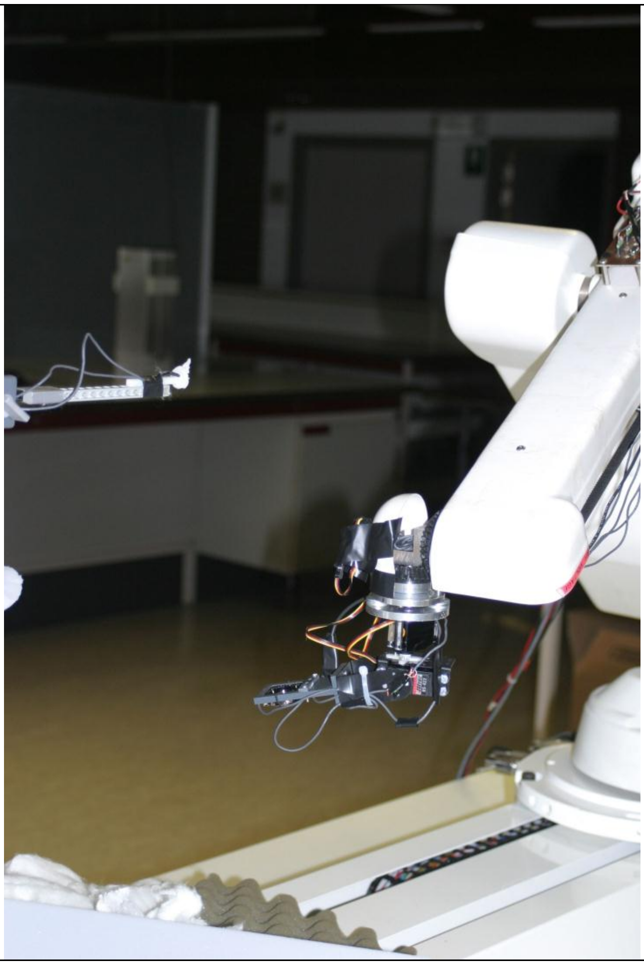

Figure 2: Picture of one of the models fixated in front of the robot arm.

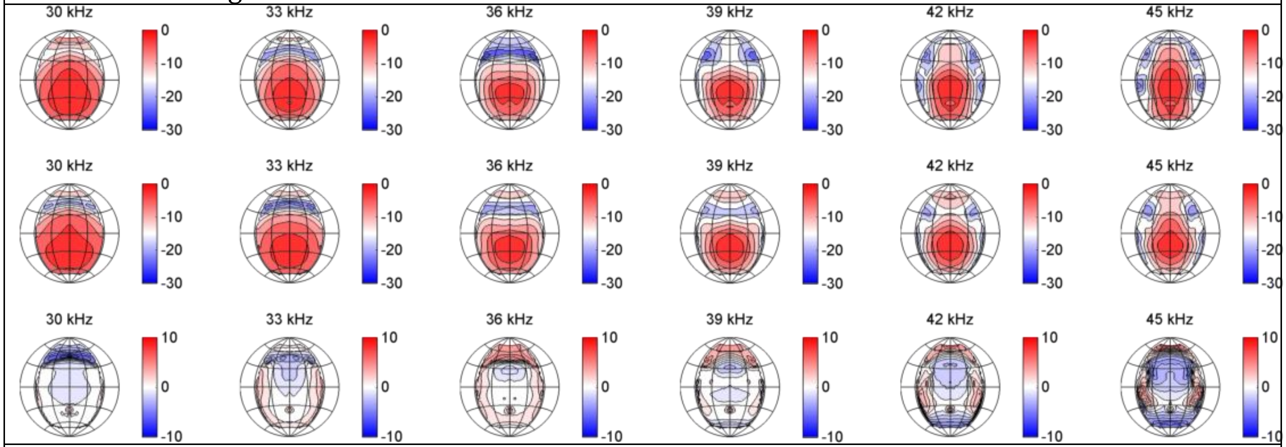

Figure 3: Resulting beam patterns for the complete model (top row) and the altered model (middle row). The bottom depicts the difference between the top and the middle row. A high resolution version of this image is available online.

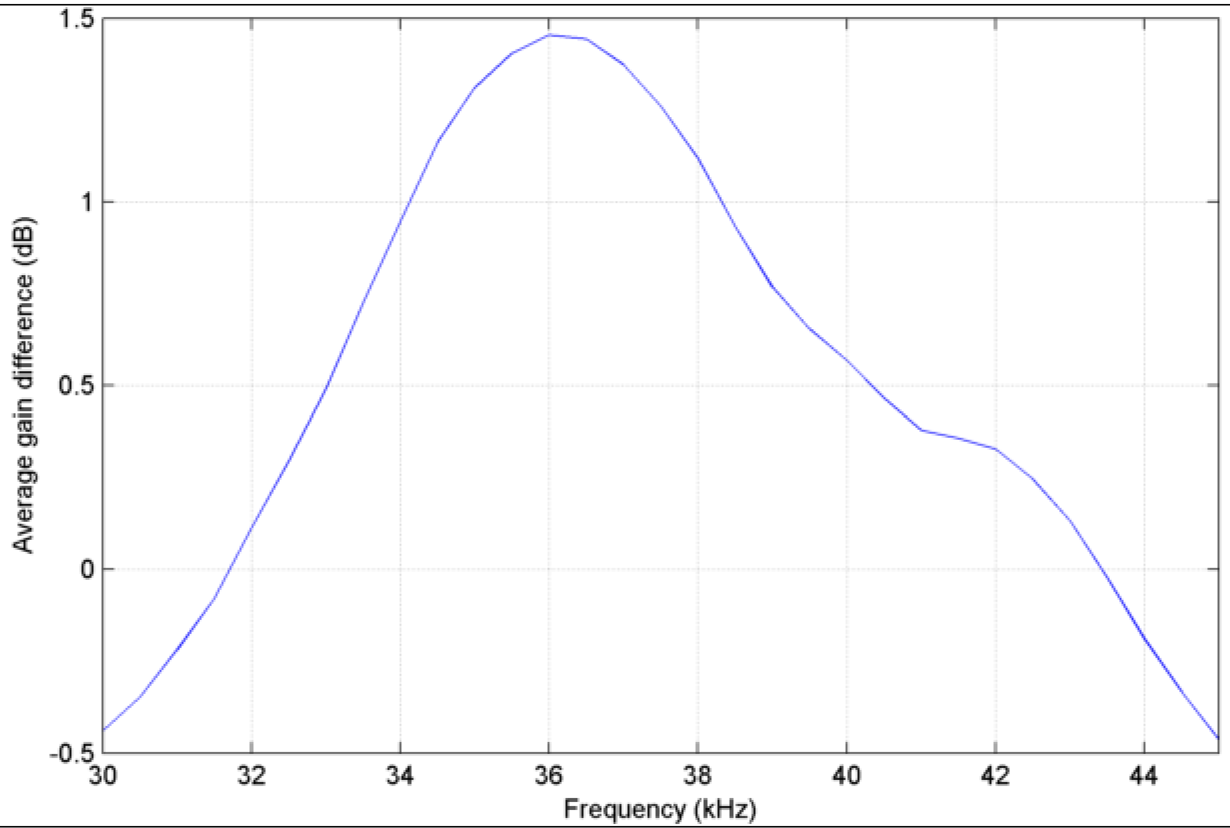

Figure 4: Effect of the flaps and the furrows as function of frequency showing the average gain difference in dB between the two models.
