# Supplementary figures and images for: The noseleaf of Rhinolophus formosae focuses the Frequency Modulated (FM) component of the calls
Source: Front Physiol. 2013 Jul 19;4:191. doi: 10.3389/fphys.2013.00191 (PMC3715718; doi:10.3389/fphys.2013.00191)

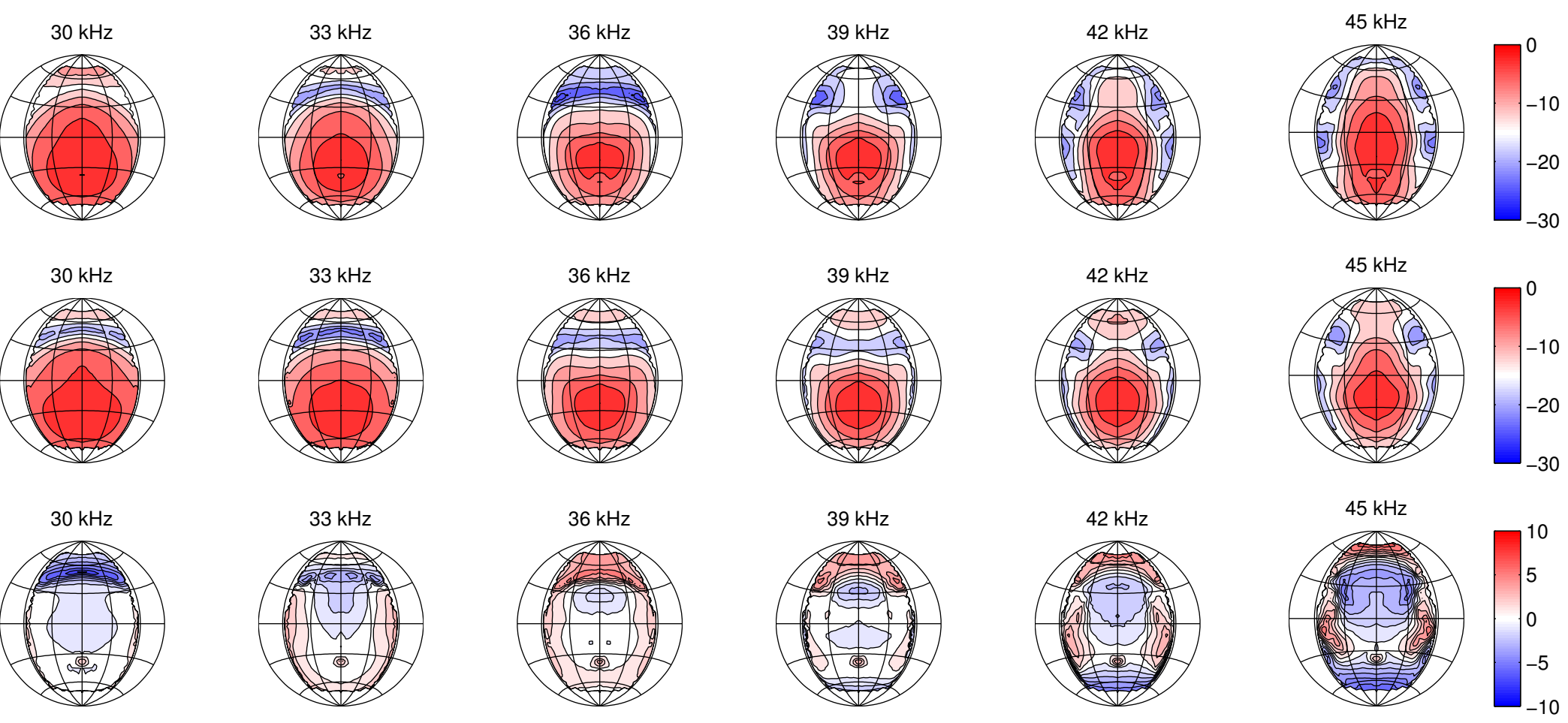

Supplement: Supplementary file 7 [file DataSheet2.PDF]
